# QTL mapping of insect resistance components of *Solanum galapagense*

**DOI:** 10.1007/s00122-018-3239-7

**Published:** 2018-11-23

**Authors:** Ben Vosman, Atiyeh Kashaninia, Wendy van’t Westende, Fien Meijer-Dekens, Henriëtte van Eekelen, Richard G. F. Visser, Ric C. H. de Vos, Roeland E. Voorrips

**Affiliations:** 1 Plant Breeding, Wageningen University and Research, PO Box 386, 6700 AJ Wageningen, The Netherlands; 2 Bioscience, Wageningen University and Research, PO Box 16, 6700AA Wageningen, The Netherlands; 3 Graduate School Experimental Plant Sciences, Droevendaalsesteeg 1, 6708 PB Wageningen, The Netherlands

## Abstract

**Key message:**

QTLs for insect resistance parameters, trichome type IV development, and more than 200 non-volatile metabolites, including 76 acyl sugars, all co-locate at the end of Chromosome 2 of *Solanum galapagense.*

**Abstract:**

Host plant resistance is gaining importance as more and more insecticides are being banned due to environmental concerns. In tomato, resistance towards insects is found in wild relatives and has been attributed to the presence of glandular trichomes and their specific phytochemical composition. In this paper, we describe the results from a large-scale QTL mapping of data from whitefly resistance tests, trichome phenotyping and a comprehensive metabolomics analysis in a recombinant inbred line population derived from a cross between the cultivated *Solanum lycopersicum* and the wild relative *S. galapagense*, which is resistant to a range of pest insects. One major QTL (*Wf*-1) was found to govern the resistance against two different whitefly species. This QTL co-localizes with QTLs for the presence of trichomes type IV and V, as well as all 76 acyl sugars detected and about 150 other non-volatile phytochemicals, including methyl esters of the flavonols myricetin and quercetin. Based on these results, we hypothesize that *Wf*-1 is regulating the formation of glandular trichome type IV on the leaf epidermis, enabling the production and accumulation of bioactive metabolites in this type of trichomes.

**Electronic supplementary material:**

The online version of this article (10.1007/s00122-018-3239-7) contains supplementary material, which is available to authorized users.

## Introduction

Insects are a major constraint in crop production. Not only do they cause direct losses by feeding, but also indirectly through the viruses they transmit (Navas-Castillo et al. 2011). Plants have developed defences to deal with pests by affecting their preference and/or performance. Defences may be constitutively present or induced upon attack. The defence mechanisms directly or indirectly affect pest settling and/or feeding behaviour and may alter their physiology, resulting in a reduced growth, development and/or reproduction, or ultimately even lead to the death of the pest (Broekgaarden et al. 2011). The direct defence system may include mechanical protection like trichomes, thorns, spines and thicker leaves and/or chemical protection based on secondary metabolites that kill or deter the herbivores or attract the insect’s natural enemies (Birkett and Pickett 2014; Glas et al. 2012; Maharijaya and Vosman 2015; War et al. 2014).

Tomato suffers from several pests and growers would benefit from resistant varieties. Resistance to insects has been identified in wild relatives of the cultivated tomato (Firdaus et al. 2012; Muigai et al. 2003; Rodriguez-Lopez et al. 2011; Silva et al. 2014; Vosman et al. 2018). In tomato and its relatives, different types of trichomes can be found, both glandular and non-glandular. Leaf trichomes were shown to play a critical role in the interaction with pests. Several lines of evidence show that the glandular trichome type IV is particularly important in resistance against insects (Firdaus et al. 2012; Muigai et al. 2003; Rakha et al. 2017a, c; Simmons et al. 2005). Trichome type IV occurs on some accessions of tomato wild relatives, including *S. pennellii*, *S. habrochaites*, *S. galapagense* and *S. pimpinellifolium.* The presence of trichome type IV is often, but not always, accompanied by resistance against whiteflies (Lucatti et al. 2013; Muigai et al. 2003; Rakha et al. 2017b). Depending on the species, glandular trichomes are able to produce/accumulate one or more classes of phytocompounds such as terpenoids, phenylpropenes, flavonoids, methyl ketones, acyl sugars and defensive proteins (Glas et al. 2012). Glandular trichomes have become a breeding target to increase insect resistance in cultivated tomato (Firdaus et al. 2013; Glas et al. 2012; Lawson et al. 1997; Smeda et al. 2018). Particularly, the acyl sugars produced in glandular trichomes may play a major role in resistance against insects (Dias et al. 2016; Liu et al. 1996; Puterka et al. 2003; Slocombe et al. 2008; Smeda et al. 2018; van den Oever-van den Elsen et al. 2016). Interactions between the effects of different metabolites on whiteflies may occur, and thus the chemical defence may only be effective when the plant can produce and accumulate a specific mixture of compounds in the trichome that is deleterious to the insect and readily released upon contact. Trichomes type I and IV can synthesize and secrete a variety of acyl sugars (McDowell et al. 2011), and some key genes involved in their synthesis and modification have been identified (Fan et al. 2016; Schilmiller et al. 2012, 2015, 2016; Smeda et al. 2018).

In *S. galapagense,* we previously identified a major QTL for whitefly (*B. tabaci*) resistance (*Wf*-1) at the end of Chromosome 2, using an F2 population derived from a cross between *S. galapagense* and *S. lycopersicum*. This QTL affected the presence of trichome type IV as well as the performance of *B. tabaci* (Firdaus et al. 2013). Analysis of metabolites in resistant and susceptible plants of the F2 population that was used for the QTL mapping showed a high accumulation of acyl sugars in resistant plants (Firdaus et al. 2013). In the present paper, we address the following questions: (1) Does the *Wf*-1 QTL identified for *B. tabaci* resistance also affect another whitefly species, *T. vaporariorum*? (2) Does the QTL also play a role in the accumulation of metabolites? (3) Do acyl sugars also map to this *Wf*-1 QTL region and are possibly other regions of the genome involved as well? To address these questions, we made use of a recombinant inbred line population that was developed based on the F2 population previously created (Firdaus et al. 2013).

## Materials and methods

### Plant materials and growing conditions

The recombinant inbred line (RIL) population is of the F7 generation and based on an F2 population derived from a cross between a TMV^®^ Moneymaker (PRI91117) and *S. galapagense* (PRI95004) (Firdaus et al. 2013). The RIL population was obtained by single seed descent. Plants were grown in 17-cm pots with potting compost in a greenhouse at 23/19 °C (day/night temp), relative humidity (RH) 70%, light 16 h/8 h (day/night) at Unifarm, Wageningen, The Netherlands. Plants were randomized in the greenhouse.

### Whitefly resistance assay and trichome type characterization

Rearings of *T. vaporariorum* and *B. tabaci* (MEAM1) were maintained on tomato cv. Moneymaker (CGN14330). Whitefly resistance assays were carried out according to (Lucatti et al. 2013) on 6-week-old plants. The *T. vaporariorum* evaluation was carried out in September (2015), and the *B. tabaci* evaluation in June (2015). Plants (one per RIL) were randomized over 2 greenhouse compartments. Each compartment contained also 5 plants of both parents. Synchronized one-day-old whiteflies were used for the experiment. Each plant received 3 clip-on cages containing 5 female whiteflies on the abaxial side of the first, second and occasionally the third fully expanded leaf. After 5 days, the number of living and dead whiteflies and the number of eggs were counted. Trichomes on the third fully expanded leaf (abaxial side) of 6-week-old plants were observed under the binocular microscope and characterized according to Luckwill (1943). During the *T. vaporariorum* evaluation, the density of trichomes type IV and V next to the midvein, in a circle marked with a Pasteur pipette, was estimated in 6 classes (0, no trichomes of the scored type; 1, less than 5; 2, 5–15; 3,15–30; 4, 30–45; 5, > 45). During the *B. tabaci* evaluation, trichome density (the number of trichomes per mm^2^) was determined by counting in three circles of 1.5 mm^2^ at each side of the midrib of the abaxial leaf surface, using a stereo microscope (40×).

### LCMS profiling of leaves

Leaflets for metabolite analysis were collected opposite of the leaflets that were used for the *T. vaporariorum* resistance assay. Leaves were flash-frozen in liquid nitrogen and stored at − 80 °C until use. Samples were processed for liquid chromatography–mass spectrometry (LC–MS) in acidified aqueous methanol as described previously (Vosman et al. 2018). A Waters Acquity HPLC coupled to a Thermo Ion Trap-Orbitrap FTMS hybrid MS system operating in negative electrospray ionization mode, a Phenomenex Luna C18 (2) column and a gradient of water and acetonitrile, both acidified with 0.1% FA, were used as described by Firdaus et al. (2013). Data processing and analysis was carried out in an essentially untargeted manner using the Metalign-MSClust workflow as described by Vosman et al. (2018), resulting in a large spreadsheet with the relative intensity of each compound detected, either known or yet unknown, in each sample. Both sucrose- and glucose-based acyl sugars with 2, 3 or 4 acyl chains were annotated based on their specific accurate masses and retention times (Vosman et al. 2018), using the mass of the formic acid adduct generated in the mass spectrometer as this [M + CH_2_O_2_–H]^−^ adduct signal was considerably higher than its original [M–H]^−^ molecular ion. Only metabolites that were detected in at least 20% of the population were considered in the QTL analysis described below.

### Genotyping and genetic map construction

The F6 generation of the RIL population, *S. lycopersicum* cv. Moneymaker and *S. galapagense* PRI95004 were genotyped using the Solcap array (Sim et al. 2012) by Trait Genetics (Gatersleben, Germany). Data were obtained for 7720 markers in 115 lines. After removing monomorphic markers, markers with missing values and markers showing a very skewed segregation in the offspring, 2836 polymorphic markers remained.

A genetic linkage map was calculated with JoinMap 4.1 (Van Ooijen 2011) using the 115 lines. SNP markers that showed an identical segregating pattern were considered as one marker. This resulted in 1253 polymorphic markers that were used to construct the genetic map. The resulting map consisted of 1095 markers divided over 12 chromosomes with 56–123 markers per chromosome, and chromosome lengths of 82–130 cM, with a total map length of 1204 cM.

### Mapping whitefly resistance and related traits in the RIL population

MapQTL 6 (Van Ooijen 2009) was used to find significant associations between molecular markers and whitefly resistance traits. The genetic linkage and QTL maps were drawn using MapChart 2.3 (Voorrips 2002). Phenotypic data were available for 111 of the 115 RIL lines.

For each of the whitefly resistance parameters, the trichome densities and a random set of 10 metabolites, LOD thresholds corresponding to a significance level of 0.05 were determined using the MapQTL permutation test with 1000 iterations. QTLs for all metabolites were tested against the average LOD threshold of the 10 random metabolites. QTL analyses of both whitefly resistance parameters and trichome densities were performed with multiple QTL mapping in order to detect any secondary minor QTL. QTL mapping of all metabolites (untargeted and acyl sugars) was performed with interval mapping in order to identify mQTL hot spots and mQTL closely linked to trichome and resistance QTL. For selected metabolites, multiple QTL mapping was performed.

QTL hot spots on the map were identified by determining the number of times a marker was located within the 2-LOD interval of a metabolite. The 2-LOD interval is used to set borders around the marker with the highest LOD score (top marker). It flanks the region most likely to contain the gene of interest. To identify it, we take the LOD score of the marker with the highest association (*x*), subtract 2 LOD units and find the nearest marker that has a LOD score ≤ *x *− 2 on either side of the top marker. (For instance, if the marker with the highest association has a LOD score of 10, the 2-LOD interval will be indicated by the nearest markers that have a LOD score ≤ 8.) These flanking markers mark the 2-LOD interval.

### Validation of metabolite QTLs in the hot spot on Chromosome 2

Recombinant inbred line R174 is heterozygous for the major metabolite QTL hot spot at the end of Chromosome 2, from solcap_snp_sl_58447 till the end. Near-isogenic lines were obtained by selecting homozygous plants in the F7 generation, using solcap_snp_sl_58447 marker, containing either the *S. galapagense* (R174A) or the cv. Moneymaker (R174B) allele. Both lines were used for the QTL validation experiment. Lines were grown in a similar way and period of the year as the whole RIL population, and leaf metabolites were detected using LCMS as described above. For each line, two bulks were made, each containing leaf material of 3 individual plants.

### Data analyses

Whitefly data: Per plant, the observations from the clip-on cages were combined. Data analysis was carried out according to Vosman et al. (2018). Survival was expressed as (living whiteflies)/(living + dead whiteflies). The numbers of eggs were divided by the estimated average number of living whiteflies present, calculated as (2*living whiteflies + dead whiteflies)/2. These data were transformed to stabilize the variances: survival as arcsine(sqrt(*x*)) and eggs as sqrt(*x*). The effects of greenhouse compartment on whitefly survival and reproduction and on trichome density were tested using the replicates of the parents and found to be non-significant for all traits; therefore, no attempt was made to correct the RIL data for compartment effects. The correlation between whitefly adult survival and oviposition rate was calculated using Pearson’s correlation coefficient. Significance of the (squared) Pearson correlations was determined using a two-sided *t* test.

Metabolite data: Data analysis was carried out essentially as described by Vosman et al. (2018). In case a metabolite was not detected in a sample, a random value between 400 and 500 (45–55% of the detection threshold value) was assigned in the data matrix. For the metabolite QTL validation experiment, metabolite data of lines R174A and B were log10-transformed before carrying out t tests (in Excel). A false discovery rate (FDR) correction was applied to correct for multiple comparisons. The corresponding *q* values were calculated according to Benjamini and Hochberg (1995). Selected metabolites were annotated by comparing the measured [M–H]^−^ accurate masses with those calculated from metabolites in public databases including KNApSAcK (http://kanaya.naist.jp/KNApSAcK/), Dictionary of Natural Products (http://dnp.chemnetbase.com), Metlin (https://metlin.scripps.edu/), HMD (http://www.hmdb.ca) and in-house libraries, within a mass deviation of 5 ppm.

## Results

### Molecular characterization of the recombinant inbred line population

The RIL population, which was obtained by single seed decent, was based on the F2 derived from a single F1 plant of the cross between a TMV^®^ Moneymaker (PRI91117) and *S. galapagense* (PRI95004) (Firdaus et al. 2013). The population was genetically characterized using the Solcap array (Sim et al. 2012). After data clean-up, 2836 polymorphic markers remained. Based on these markers, the average percentage homozygosity was 96.0% (85.6–99.7%) for the RIL lines, 99.4% for PRI91117 and 95.3% for PRI95004.

### Mapping whitefly resistance and trichome traits

**Fig. 1:**
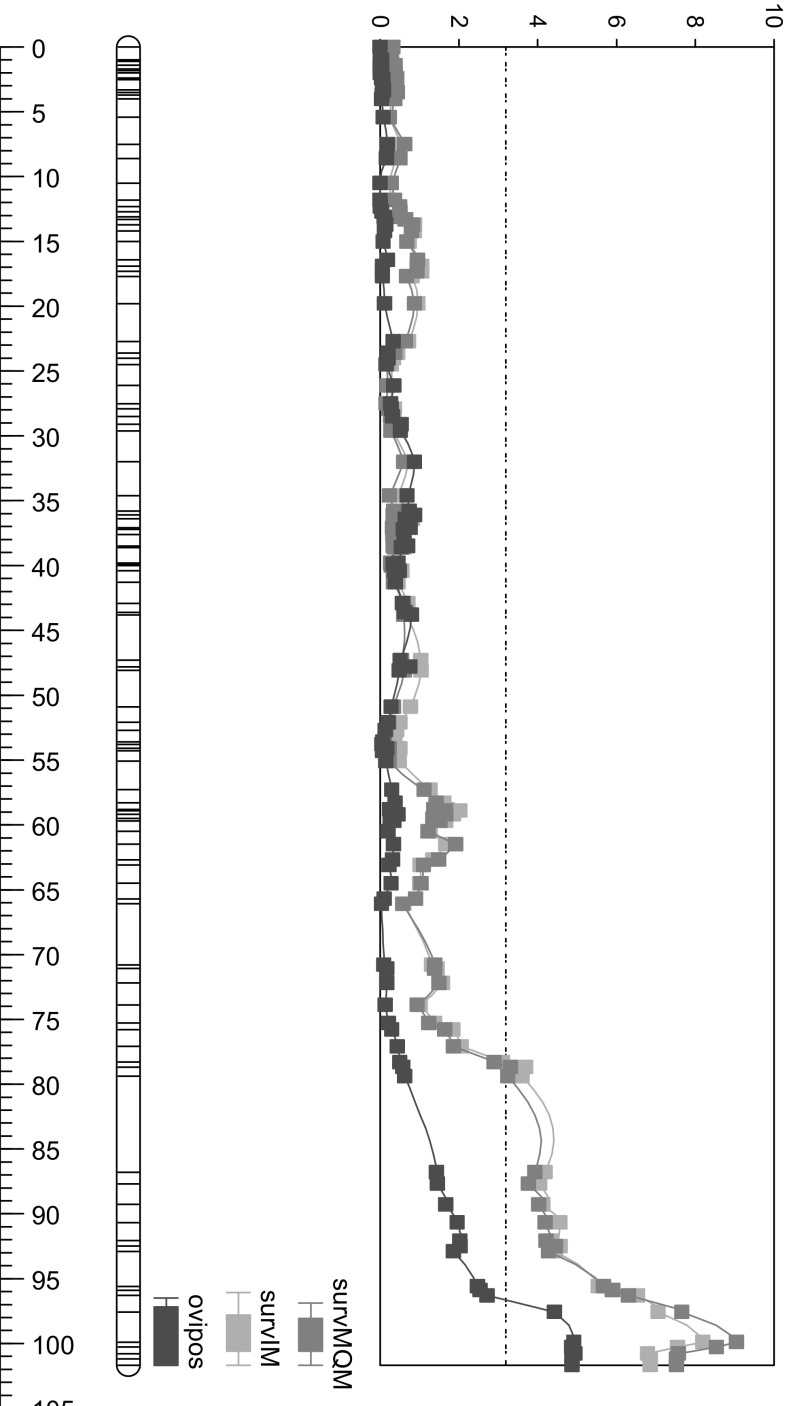
LOD profile for the *T. vaporariorum* resistance traits mapping to Chromosome 2. Right bar indicates the genetic distance in Cm. Top scale indicates LOD score. Red lines indicate whitefly adult survival (light red = interval mapping, dark red = MQM mapping), and blue line whitefly oviposition (colour figure online)

The RIL population was phenotyped for *T. vaporariorum* resistance (both adult survival and oviposition rate) and *B. tabaci* (adult survival only). The same population was also phenotyped for their trichome characteristics: presence or absence of trichomes type IV and V) during the *T. vaporariorum* evaluation, and density of trichomes type IV and V during the *B. tabaci* evaluation (Table 1). The table shows a major QTL at the end of Chromosome 2 for adult survival of both whiteflies, and the 2-LOD interval of the QTL for *B. tabaci* falls within the QTL for *T. vaporariorum*. At the same position, also QTLs are found for both the presence/absence and density of trichomes type IV and V. Presence of the *S. galapagense* allele at the end of Chromosome 2 results in production of type IV trichomes, whereas the *S. lycopersicum* allele results in type V trichomes. Figure 1 shows the LOD profile for the traits mapping to Chromosome 2. Additional minor QTLs were found on Chromosomes 5, 9 and 10. For *T. vaporariorum* oviposition rate, 3 QTLs were found, each explaining around 15% of the variance. On Chromosomes 2 and 10, the *S. lycopersicum* allele increased oviposition, whereas the *S. lycopersicum* allele on Chromosome 5 decreased oviposition (Table 1). The same Chromosome 5 region with the *S. galapagense* increased the survival of *B. tabaci*, although with a (very) low percentage of explained variance. The LOD scores and percentage explained variance for *B. tabaci* adult survival (LOD 29.1, explained variance 61.2%) were markedly higher than the values obtained for *T. vaporariorum* (LOD 8.2, explained variance 27.2%). The presence of trichome type IV shows a negative correlation with the presence of trichome type V (− 0.77) and with both adult survival (− 0.62) and oviposition rate (− 0.44) *of T. vaporariorum*.

**Table 1:** QTLs for whitefly resistance and trichome characteristics. (A) QTLs for *T. vaporariorum* adult survival (AS) and oviposition rate (OR) as well as trichomes type IV and V presence/absence; (B) QTLs for *B. tabaci* adult survival (AS) and trichomes type IV and V densities. Results in (A) and (B) are derived from two independent evaluations of the recombinant inbred lines. Indicated are the chromosome (Chrom.) on which the QTL is found, its position on the genetic map, the LOD score, explained variance (% Expl.), additive effect (Add.eff; in which a positive value indicates that the *S. lycopersicum* allele increases the trait and a negative value that the *S. galapagense* allele increases the trait), the Solcap marker (solcap_snp_sl_xxxxx) on the top locus and the 2-LOD interval (tomato genome version SL3.0)

Trait	Chrom.	Position	LOD	% Expl.	Add.eff.	LOD peak SNP	2-LOD interval SL3.0	
(A) *T. vaporariorum*								
AS	2	100.1	8.2	27.2	0.28	sl_58447	54,008,284	55,395,282
AS	10	40.6	3.2	9.7	0.17	sl_33007	58,669,819	61,400,465
OR	2	101.1	5.3	15.9	2.1	sl_32389	54,088,059	55,395,282
OR	5	58.4	5.4	16.3	− 2.2	sl_50925	6,036,748	58,896,263
OR	10	28.0	4.3	12.6	1.9	sl_62952	3,006,916	58,669,819
Tri IV^1^	2	100.1	45.6	83.7	− 1.95	sl_58447	54,454,570	54,756,547
Tri IV	9	39.2	4.3	2.8	− 0.36	sl_39667	2,142,996	4,063,393
Tri V^1^	2	100.1	13.5	37.1	1.01	sl_58447	54,088,059	55,002,829
Tri V	9	49.8	3.4	7.4	0.46	sl_54043	4,910,609	65,138,084
Tri V	10	51.4	3.0	6.5	0.44	sl_33144	59,466,180	62,404,007
(B) *B. tabaci*								
AS	2	100.1	29.1	61.2	0.55	sl_58447	54,454,570	55,002,829
AS	5	54.0	4.0	4.4	− 0.15	sl_100157	4,035,943	7,353,029
AS	10	52.6	4.5	5.1	0.16	sl_14908	61,164,437	62,404,007
Tri IV^1^	2	100.1	20.2	52.5	− 13.9	sl_58447	54,454,570	55,395,282
Tri IV	9	39.3	4.5	8.4	− 5.5	sl_100157	2,142,996	4,511,974
Tri V^1^	2	100.6	14.1	43.6	20.2	sl_20049	54,454,570	55,395,282
Tri V	9	32.0	3.9	9.4	9.4	sl_39806	1,836,627	4,063,393

^1^Trichomes type IV and V: in the *Trialeurodes* experiment (A), the density of trichomes was scored in classes (0 to 5), and in the *Bemisia* experiment (B) the number of trichomes per mm^2^ was scored

### Mapping leaf metabolites in the recombinant inbred line population

**Fig. 2:**
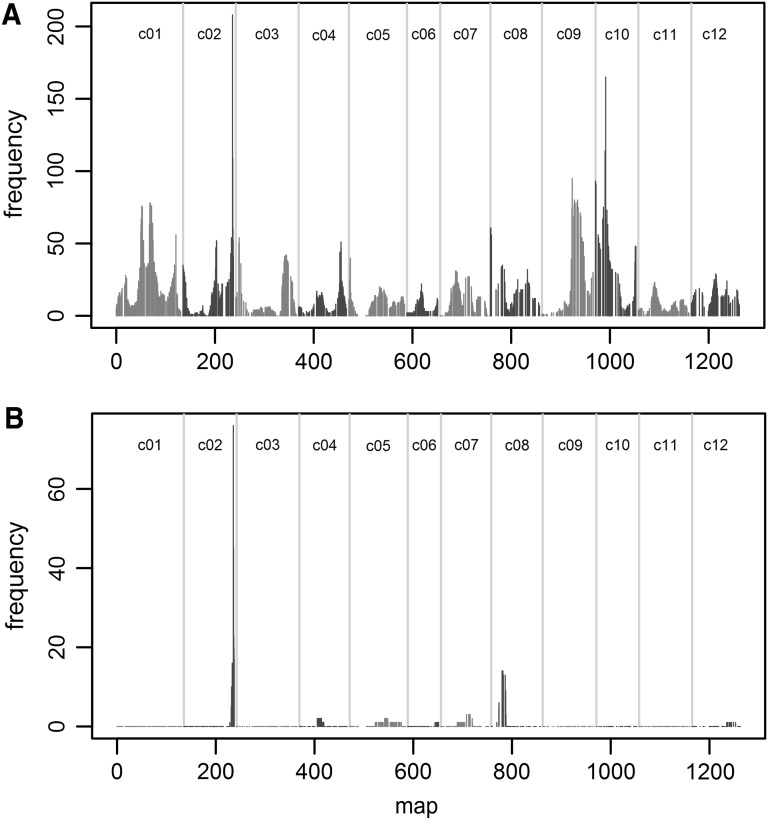
Frequency of QTLs on the tomato map. Map size on the x-axis is indicated in centiMorgan. Chromosomes are separated by vertical grey lines, and chromosome numbers are shown at the top. For each marker, the number of times it falls within a 2-LOD interval of a QTL is indicated. A: all metabolites; B: acyl sugars

A high mass resolution LC-Orbitrap FTMS-based untargeted metabolomics profiling approach was carried out for phytochemical phenotyping of the RIL population. The extraction and analysis method applied is particularly suitable for the detection of secondary metabolites including phenolic compounds, flavonoids, alkaloids, saponins and acyl sugars. Subsequent analysis of the LCMS raw data was performed in two ways: (1) untargeted using the in-house MetAlign-MSClust workflow and (2) targeted towards all possible S2–S4 and G2–G4 acyl sugars based on their specific accurate masses. Using the untargeted data processing approach, relative abundance data of 1877 metabolites were extracted (additional table S1 “All metabolites RIL”). In the targeted approach, 76 different sucrose-based acyl sugar structures, mainly with 3 or 4 acyl chains, were detected, while glucose-type acyl sugars were not detected in this population (additional table S2 “acyl sugars RIL”). QTL analyses were performed for all individual compounds from the targeted and untargeted metabolite data sets. For 1345 out of the 1877 metabolites detected in the untargeted analysis (71%), one or more QTLs were found, resulting in a total of 1946 QTLs (additional table S3 “all metabolite QTL”), while for all 76 acyl sugars one or more QTLs were detected, resulting in 99 acyl sugar QTLs (additional table S4 “acyl sugar QTL”). Figure 2 shows the distribution of the QTLs over the different chromosomes for all metabolites (A) and for acyl sugars (B). From Fig. 2a, it is clear that these metabolite QTLs are not randomly distributed over the genome of tomato: hot spots (> 50 QTLs) were found on several chromosomes, with the largest one at the end of Chromosome 2 containing 208 QTLs (11% of all metabolites). There were 215 metabolites with a 2-LOD QTL interval that overlaps the 2-LOD interval for *T. vaporariorum* adult survival on Chromosome 2 (54,008,284–55,395,282); 157 of them had a LOD score of > 10.

All 76 acyl sugars have a QTL on Chromosome 2, resulting in a strong clustering of acyl sugar QTLs (Fig. 2b). Sixty-nine of them have solcap_snp_sl_58447 as top marker and a 2-LOD interval between position 54,454,570 and 54,756,547, identical to the 2-LOD interval for trichome type IV. The remaining seven acyl sugars were associated with markers close to solcap_snp_sl_58447. The 2-LOD interval of all acyl sugar QTLs on Chromosome 2 overlaps with the 2-LOD interval of whitefly adult survival. Nineteen acyl sugars have one or more additional QTLs, with a maximum of 4 for the acyl sugar type S3:13 (4, 4, 5), i.e. sucrose with 3 acyl chains of 4, 4 and 5 carbons. Of the 21 additional QTLs, 14 are located on Chromosome 8 of which 6 are associated with the solcap_snp_sl_5428, and their 2-LOD intervals are flanked by markers on position 1,916,141 and 3,609,123 on Chromosome 8.

To validate the metabolite QTLs located in the hot spot on Chromosome 2, we constructed two near-isogenic lines containing either the *S. galapagense* allele (R174A) or the *S. lycopersicon* cv. Moneymaker allele (R174B), from solcap_snp_sl_58447 till the end of Chromosome 2. Line R174A produced glandular trichomes type IV, whereas R174B did not. Metabolites in leaves of both lines were determined using the same LCMS profiling approach as described above for the RIL population, resulting in the detection of 1237 metabolites (additional table S5 “Comparing R174AB”). There were 183 metabolites (14.7%) that differed significantly (FDR < 0.05) between R174A and 174B. Comparison of masses and retention times of these differential metabolites with those of the 215 metabolites that mapped in the RIL-QTL for *T. vaporariorum* adult survival resulted in the identification of 73 identical metabolites, of which 39 could be annotated. These mostly included acyl sugars, but also methylated forms of the flavonols myricetin and quercetin.

### Correlation of resistance traits with metabolites

Correlation analysis shows that the two *T. vaporariorum* resistance traits (adult survival and oviposition rate) were strongly correlated (correlation coefficient = 0.74). Both traits were negatively correlated with the presence/absence of type IV trichomes. For adult survival, the correlation coefficient with the presence of type IV trichomes was − 0.62, while for oviposition rate it was − 0.44. There were 114 metabolites from the untargeted analysis that showed a correlation coefficient > 0.8 with the presence of type IV trichomes, including several acyl sugars (additional table S6 “correlation analysis”). Similarly, we found 123 metabolites from the untargeted analysis that showed a correlation coefficient below − 0.6 with whitefly adult survival, again several of them were identified as acyl sugars. Table 2 shows the top 20 of metabolites that best correlate with whitefly adult survival: metabolites that show a strong negative correlation with whitefly adult survival have a strong positive correlation with the presence of type IV trichomes and a negative correlation with type V trichomes. All these metabolites map to the end of Chromosome 2.

**Table 2:** Top 20 metabolites most strongly correlating with *T. vaporariorum* adult survival (AS). Also shown are their correlations with whitefly oviposition (OR), presence of type IV trichomes (T4) and type V (T5). Details on the metabolites can be found in additional table S6 “correlation analysis”. Metabolite IDs starting with S refer to sucrose acyl sugars, while the number behind the S and the number behind the semicolon refers to the number of acyl chains and total number of carbon atoms in all acyl chains together, respectively. For instance, S3:20 refers to sucrose with 3 acyl chains that together contain 20 carbon atoms

Metabolite ID	Putative[M–H]^−^ ion	Putative ID	AS	OR	*T*4	*T*5
1222_1	737.3237	S5:20	− 0.744	− 0.587	0.904	− 0.727
655_2	813.3918	Unknown	− 0.733	− 0.586	0.923	− 0.775
1297_1	734.3243	Unknown	− 0.729	− 0.588	0.918	− 0.76
632_2	759.7676	Unknown	− 0.728	− 0.586	0.929	− 0.765
610_2	795.4020	Unknown	− 0.727	− 0.587	0.883	− 0.738
519_2	739.3394	Unknown	− 0.727	− 0.555	0.95	− 0.762
613_2	737.3610	S3:20	− 0.727	− 0.571	0.939	− 0.788
1050_1	597.2407	S3:12	− 0.726	− 0.54	0.943	− 0.732
691_1	611.2193	S4:12	− 0.726	− 0.557	0.931	− 0.814
574_2	723.3084	S5:19	− 0.722	− 0.57	0.959	− 0.765
1411_1	695.3506	S3:19	− 0.722	− 0.582	0.916	− 0.725
1376_1	781.3864	Unknown	− 0.72	− 0.584	0.938	− 0.75
1587_1	807.4391	S4:26	− 0.718	− 0.555	0.933	− 0.714
1487_1	765.3914	S4:23	− 0.716	− 0.575	0.849	− 0.706
329_2	611.2195	S4:12	− 0.714	− 0.573	0.816	− 0.81
1406_1	723.3450	S4:20	− 0.712	− 0.562	0.932	− 0.775
1391_1	667.3551	S2:18	− 0.711	− 0.569	0.908	− 0.713
1086_1	702.2616	Unknown	− 0.711	− 0.553	0.89	− 0.766
1579_1	793.4235	S4:25	− 0.71	− 0.544	0.961	− 0.741
1419_1	781.3865	Unknown	− 0.709	− 0.591	0.91	− 0.769

## Discussion

### A metabolite QTL hot spot co-localizes with the trichome type IV QTL

The leaf metabolome of *S. galapagense* differs strongly from that of *S. lycopersicum*, both in the presence/absence of metabolites and in their relative abundance (Lucatti et al. 2013; Vosman et al. 2018). This difference allowed the mapping of QTLs for 1345 out of the 1877 leaf metabolites detected by untargeted, high mass resolution LCMS analysis of a RIL population derived from an inter-species cross. These metabolite QTLs (mQTLs) were not randomly distributed over the tomato genome, but showed a clear clustering resulting in hot spots and cold spots. We detected several hot spots with more than 50 mQTLs (Fig. 2), the strongest one with more than 200 mQTLs at the end of Chromosome 2. Identifying hot spots and cold spots for mQTLs in plant genomes is not uncommon (Carreno-Quintero et al. 2012; Keurentjes et al. 2006; Maharijaya et al. 2018; Rowe et al. 2008; Wahyuni et al. 2014). In a *Solanum pennellii* introgression population, hot spots and cold spots were detected for mQTLs related to secondary metabolism in tomato fruit pericarp (Alseekh et al. 2015), while in pepper fruits two closely linked mQTL hot spots were detected that regulated the relative levels of 35 and 103 secondary metabolites, respectively (Wahyuni et al. 2014). In leaves of the same pepper population, three mQTL hot spots were detected, of which one overlapped with the fruit mQTL hot spots (Maharijaya et al. 2018). Clustering of mQTLs may point at a common regulator and/or involvement in the same biochemical process (Fu et al. 2009). In the present work on tomato leaves, metabolites that make up the QTL hot spot on Chromosome 2 are highly correlated with the presence of trichome type IV. In addition, their QTL positions co-localize with the major QTL for trichome type IV presence/density, strongly suggesting that these metabolites are in fact produced or accumulated in the trichomes type IV. Based on these observations, we hypothesize that the common regulatory process is the ability to form glandular trichome type IV on their leaves. Once the glandular head is formed, a large number of phytochemicals, including acyl sugars, can be produced and accumulated.

### Resistance against whiteflies in *Solanum galapagense* is for a large part controlled by the *Wf*-1 QTL

A major QTL at the end of Chromosome 2 of *S. galapagense* was previously shown to confer resistance against the whitefly *B. tabaci* (Firdaus et al. 2013). In the present paper, we confirm that result and in addition show that the QTL confers resistance to the greenhouse whitefly *T. vaporariorum* as well. The fact that the survival of both whitefly species is affected by the same QTL strongly suggests the same mechanism underlying the resistance. The 2-LOD interval of 215 metabolite QTLs, 16% of all metabolites that could be mapped, overlaps with the QTL for whitefly adult survival, suggesting that either one or a combination of these metabolites is responsible for the observed effect on the whiteflies. Among these co-localizing metabolites, 76 acyl sugars are detected and half of them have a negative correlation (corr. coeff. < −0.6) with whitefly adult survival. In fact, 72 out of the 76 acyl sugars have a highly significant correlation with whitefly adult survival (*P* < 0.001, which is similar to a correlation coefficient of < −0.32). Acyl sugars may negatively affect insect and mite survival, as was shown previously (Luu et al. 2017; Puterka et al. 2003). Resistance based on acyl sugars may also depend on the complexity and abundance of the acyl sugar mixture (Leckie et al. 2016). Currently, we cannot conclude whether the observed whitefly resistance is due to the presence of one specific metabolite, out of the total of 215 co-localizing metabolites, or a blend of these metabolites. The acyl sugars identified are among the most obvious candidates, but the methyl esters of the flavonols myricetin and quercetin, which co-accumulated and co-correlated with the acyl sugars in the glandular trichomes type IV, are good candidates as well (Morant et al. 2003; Treutter 2005; Vosman et al. 2018). The QTL region for whitefly adult survival contains several genes putatively involved in the biosynthesis of these compounds (Vosman et al. 2018). Further research is needed to elucidate their role.

The LOD score (29.1) and percentage explained variance (61.2) for *B. tabaci* adult survival in the RIL population were similar to those measured in the F2 population of this *S. galapagense* x *S. lycopersicon* cross (LOD 28.1, explained variance 54.1) (Firdaus et al. 2013). Interestingly, this LOD score is markedly higher than the value obtained with *T. vaporariorum* (LOD: 8.2). This indicates that, although the same QTL is found to affect adult survival of both whitefly species, there may be more experimental noise in the survival data of *T. vaporariorum*. A possible cause for this additional noise may be that *T. vaporariorum* is stronger than *B. tabaci,* making it occasionally possible for *T. vaporariorum* to escape from the leaf even though it was “glued” by the sticky content of the type IV trichomes (Rakha et al. 2017a; Vosman et al. 2018). Alternatively, some compounds related to the resistance may have a more detrimental effect on *B. tabaci* than on *T. vaporariorum*.

Next to the *Wf*-1 QTL on Chromosome 2, a second QTL affecting whitefly adult survival and oviposition rate (called *Wf*-2) was detected on Chromosome 9 in the F2 population (Firdaus et al. 2013). This QTL was not found back in the RIL population with either whitefly species. Firdaus et al. showed that the effect of the *Wf*-2 QTL was only detected in plants that were heterozygous for the *Wf*-1 QTL. Such plants are almost completely absent in our RIL population, which may explain why we did not detect this *Wf*-2 effect. In the RIL population, we detected new minor QTLs for whitefly adult survival on Chromosomes 5 (only *B. tabaci*) and 10 (both species) (Table 1), which only explain a low percentage of the variance (< 10%) and need further validation. Interestingly, for the minor effect QTL (explained variance only 4.4%) on Chromosome 5, the allele that reduces *B. tabaci* adult survival was inherited from the *S. lycopersicum* parent. This QTL does not (significantly) affect *T. vaporariorum* survival. For oviposition of *T. vaporariorum,* QTLs were detected on the Chromosomes 2, 5 and 10, each explaining a similar part of the variance (approx. 15%). The QTL on Chromosome 2 co-localizes with *Wf*-1 QTL for adult survival. The Chromosome 5 QTL overlaps with the *B. tabaci* adult survival QTL, and like for that QTL the allele that confers increased resistance (reduced oviposition in this case) was inherited from the *S. lycopersicum* parent. It remains to be seen if both traits mapping to Chromosome 5 (*B. tabaci* adult survival and *T. vaporariorum* oviposition rate) involve the same gene. The Chromosome 10 QTL for oviposition of *T. vaporariorum* overlaps with the metabolite hot spot on Chromosome 10, which may be responsible for the effect.

### Role of *Wf*-1 QTL in acyl sugar synthesis

All acyl sugars detected had a QTL at the end of Chromosome 2 which perfectly overlaps with the QTLs for trichome type IV presence and density. Using the near-isogenic lines R174A (*S. galapagense* allele of *Wf*-1) and R174B (*S. lycopersicum* allele of *Wf*-1), we could show that plants containing the *S. galapagense* allele indeed produced type IV trichomes, whereas the plants with the *S. lycopersicum* allele did not, clearly indicating that this *Wf*-1 QTL regulates trichome type IV development. Using metabolite analysis of the same near-isogenic lines, we were also able to validate about half of the mQTLs that were overlapping with the *Wf*-1 QTL, including a large number of acyl sugars. The observation that not all mQTLs could be validated may be due to the fact that some metabolites for which a QTL was found were not present or below detection level in both lines R174A and R174B, while others did not differ significantly at the FDR threshold level. The fact that all identified acyl sugar QTLs co-localize with *Wf*-1 fits with the presumed key role of *Wf*-1 in regulating the presence or density of trichome type IV in which the acyl sugars are assumed to be produced and accumulated. However, we cannot exclude that the acyl transferase present in the QTL region, i.e. Solyc02g093180, may play a role as well, as this type of gene may be involved in acyl sugar biosynthesis (Fan et al. 2016). For a small number of acyl sugars, additional minor QTLs were found as well. The 14 acyl sugar QTLs on Chromosome 8 have a relatively low LOD score (3.3–5.8) and explained variance (12.7–21.6%) compared to the Chromosome 2 QTLs. All except one have a 2-LOD interval in which one candidate gene, i.e. Solyc08g013830, with an IPR003480 acyl transferase domain which is putatively involved in acyl sugar production (Fan et al. 2016), is present. So far, both acyl transferases have not been studied in detail and their possible role in whitefly resistance warrants further research. Acyl sugar biosynthetic genes (ASAT1 through 4) that have been characterized previously (Fan et al. 2016; Schilmiller et al. 2012, 2015) are not found in any of the minor QTL regions, except ASAT2, which may be underlying the minor QTLs identified on Chromosome 4. Apparently, there is no genetic variation for these genes present among the parents of the population used and the structural genes needed for acyl sugar production are present in the cultivated tomato. While acyl transferase genes are needed for the production of acyl sugars (Fan et al. 2016; Schilmiller et al. 2012, 2015), a common regulatory (transcription) factor located in the Wf-1 QTL may be responsible for the increased levels of acyl sugars in *S. galapagense*. It is interesting to note that also acyl sugar-rich tomato line CU071026, which contains 5 introgressions from *S. pennellii*, has an introgression at the bottom of Chromosome 2 that is necessary for high levels of acyl sugar production (Leckie et al. 2012). It may be speculated that homologous genes are involved.

## Conclusions and perspective

We have shown that the major QTL at the end of Chromosome 2 of *S. galapagense* is involved in the resistance against two whitefly species. This QTL regulates the production of glandular trichome type IV as well as the production of a large number of metabolites including all detected acyl sugars, which are frequently associated with whitefly resistance. As the effect of this QTL is pleiotropic, it is likely that the causal gene possibly is a transcription factor controlling the formation of the glandular head of the trichome. However, it cannot be excluded that other genes present in the QTL region are also responsible for the resistance. Recently, we have shown that *S. galapagense* is resistant to a range of insects including, next to *T. vaporariorum* and *B. tabaci,* also *Myzus persicae, Frankliniella occidentalis* and *Spodoptera exigua* (Vosman et al. 2018). Considering the mode of feeding of these insect species, it is conceivable that the trichome-based resistance mechanism of *S. galapagense* is effective against aphids and thrips as well. Possibly, the ability to form glandular trichomes IV and thereby its specific metabolites, including acyl sugars, was lost during domestication of tomato, resulting in susceptibility towards insects of the cultivated material (McDowell et al. 2011). Reintroducing trichome IV production into tomato will most likely restore insect resistance.

### Author contribution statement

BV and REV conceived and designed the experiments. FM produced the RIL population. WvW and AK carried out the whitefly experiments with Tv and Bt, respectively. HvE and RdV carried out the metabolite analysis and compound annotation. RdV, REV and BV carried out data analysis and produced the first draft of the manuscript. AK and RGFV were involved in revising the manuscript.

## Electronic supplementary material

Below is the link to the electronic supplementary material.

Supplementary Table S1: All metabolites detected in the LCMS analysis of the RIL population. Supplementary Table S2: All acyl sugars detected in the LCMS analysis of the RIL population. Supplementary Table S3: All metabolite QTLs detected in the LCMS analysis of the RIL population. Supplementary Table S4: All acyl sugar QTLs detected in the LCMS analysis of the RIL population. Supplementary Table S5: All metabolites detected in lines R174A and R174B. Supplementary Table S6: Correlation of all metabolites with *T. vaporariorum* adult survival (AS), oviposition (OR) and trichome type IV and type V presence. (XLSX 2491 kb)
